# Raman scattering and vacuum fluctuation: An Einstein-coefficient-like equation for Raman cross sections

**DOI:** 10.1063/5.0171382

**Published:** 2023-11-15

**Authors:** Wei Min, Xin Gao

**Affiliations:** Department of Chemistry, Columbia University, New York, New York 10027, USA

## Abstract

Since it was first predicted 100 years ago, Raman scattering has been a cornerstone of molecular spectroscopy with a widespread impact on science and technology. Nearly all theoretical frameworks have employed Raman cross sections (σ_Raman_) to characterize and quantify molecular Raman response. The recently introduced absolute stimulated Raman scattering cross section (σ_SRS_), on the other hand, provides an alternative way of interpreting molecular responses under two coherent laser sources. However, the theoretical connection between σ_Raman_ and σ_SRS_ remains unclear. Herein, we are inspired by Einstein’s A and B coefficients for spontaneous and stimulated emissions and derived an analogous equation [Eq. (16)] for Raman scattering from an approach along quantum electrodynamics. Equation (16) decomposes Raman cross sections into a contribution from the vacuum electromagnetic field and an underlying molecular response captured by stimulated Raman cross sections (in the unit of Göppert–Mayer). This theoretical relation is supported by recent experimental measurements on methanol as a model compound. Foremost, it provides a connection between experimentally defined σ_Raman_ and σ_SRS_ under certain approximations. In addition, it quantitatively shows that it is the weak vacuum field of the Stokes channel that makes Raman cross sections appear so small, corroborating the conventional Raman theory. Moreover, it suggests stimulated Raman cross sections to be a vacuum-decoupled intrinsic quantity for characterizing molecular response during Raman scattering. Remarkably, stimulated Raman cross sections turn out to be not weak when compared to two-photon absorption, narrowing the conventional gap of cross sections between spontaneous Raman and UV–vis absorption by more than 10^10^ folds.

## INTRODUCTION

Raman scattering, an inelastic light scattering process by molecules, is a cornerstone of molecular spectroscopy with a wide impact on physical science, life science, and technology. While the effect was first experimentally observed by Raman and Krishnan in 1928,^1^ it was first theoretically predicted by Smekal in 1923.^2^ In fact, it has been referred to as the Smekal–Raman–Effekt in older German-language literature.^3–5^ Hence, 2023 marks the 100th anniversary of Raman scattering.

All Raman literature studies have employed Raman cross sections, σ_Raman_, as a measure of the molecular response for the interaction strength between light and molecule.^6^ The rate of energy (unit in J/s) scattered into the Stokes channel (considered to be a differential rate into solid angle dΩ), P_Raman_, is written as the product of the number of vibrational modes, n, differential Raman cross section (unit in cm^2^) of the mode, dσ_Raman_, with the incident intensity [unit in J/(s·cm^2^)] of the pump beam,

$$\text{d}{\text{P}}_{\text{Raman}}=\text{n}\cdot \text{d}\sigma_{\text{Raman}}\;\cdot {\text{I}}_{\text{pump}},$$ (1)

where σ_Raman_ can be theoretically expressed with other spectroscopic quantities from quantum mechanics. Popular theoretical frameworks include Kramers–Heisenberg formulation derived from the second-order perturbation theory or third-order perturbation expansion of the density matrix operator.^7,8^ The effect of vacuum electromagnetic field is implicitly treated in most of these frameworks.

Very recently, a new framework was proposed to understand molecular response under stimulated Raman scattering (SRS),^9^ a process closely related to the classic Raman scattering. SRS spectroscopy and microscopy have made an increasingly broad impact on ultrafast spectroscopy, analytical science, and biological imaging.^10–15^ SRS literature has also been relying on σ_Raman_ for decades.^13,16–18^ To quantify the response of the emerging Raman-active imaging probes, absolute stimulated Raman cross section, σ_SRS_, was recently introduced, carrying a unit of Göppert–Mayer (1 GM = 10^−50^ cm^4^ ⋅ s ⋅ photon^−1^).^9^ A systematic comparison between the measured σ_SRS_ and the reported two-photon absorption cross sections for real molecules has provided useful insights. While σ_SRS_ was phenomenologically introduced to describe molecules under SRS excitation (i.e., strong pump and strong Stokes beams), we hypothesize that its significance can extend beyond SRS experiments, as it might provide the vacuum-field-independent molecular response for all Raman scattering processes. However, an important question needs to be addressed: is stimulated Raman cross section compatible with the traditional framework of Raman scattering [Eq. (1)]? If so, can σ_Raman_ and σ_SRS_ of the same molecular mode be related to each other?

Herein, we attempt to connect σ_Raman_ and σ_SRS_ without referring to the full quantum theory. The physical picture is to treat normal Raman as a stimulated Raman process driven by zero-point energy of vacuum electromagnetic field as the Stokes beam, an accepted view in quantum electrodynamics (QED) theory.^19^ In doing so, we have derived an Einstein-coefficient-like relation, Eq. (16), which decomposes σ_Raman_ into a contribution from the vacuum field and an underlying molecular response captured by σ_SRS_. This relation [Eq. (16)] is analogous to the Füchtbauer–Ladenburg equation describing fluorescence. This relation is interesting in several aspects. Foremost, it provides a connection between experimentally defined σ_Raman_ and σ_SRS_ under certain approximations. In addition, the agreement with experimental measurement supports that it is indeed plausible to think of spontaneous Raman as stimulated Raman driven by zero-point radiation of the vacuum. Moreover, this relation provides a quantitative perspective to reconcile the apparently feeble Raman cross sections. In short, it is the weak vacuum field, as calculated to be the equivalent of a propagating beam of microwatt power level, that makes Raman cross sections so small. Furthermore, Eq. (16) also suggests that σ_SRS_ is a vacuum-decoupled intrinsic quantity for describing molecular Raman response, given that the vacuum state can be altered in cavity QED.^20–23^ When compared with other nonlinear optical processes, this molecule-intrinsic Raman response is not weak after all, narrowing the gap of conventional comparison of cross sections by over 10^10^ folds.

## RESULTS

### Definitions of Raman cross sections and stimulated Raman cross sections

We begin by comparing the definitions of the two sets of cross sections. Equation (1) defines the commonly utilized Raman cross section σ_Raman_ found in the literature. Note that σ_Raman_ is defined in terms of energy flux due to historic reasons, rather than photon flux, a unit more commonly used in many other optical processes. We can rewrite Eq. (1) in terms of photon flux as

$$\text{d}{\text{R}}_{\text{Raman}}=\text{n}\cdot \text{d}\sigma_{\text{Raman}}\cdot \phi_{\text{pump}}\cdot \frac{\hbar \omega_{\text{p}}}{\hbar \omega_{\text{S}}},$$ (2)

where ℏω_p_ and ℏω_S_ are the energy of the pump and Stokes photons, respectively, dR_Raman_ represents the generation rate of Stokes photons into solid angle dΩ, in the unit of photon ⋅ s^−1^, and *ϕ*_pump_ is the photon flux of the pump beam, in the unit of photon/(s ⋅ cm^2^). Note that dR_Raman_ also describes the rate of vibrational transition of the target vibrational mode that accompanies the differential photon generation, as each Stokes photon generation is accompanied by a quantum transition to the vibrational excited state of the mode.

Inspired by the theory of two-photon absorption, a new framework has been recently introduced to define absolute stimulated Raman cross section, σ_SRS_, for a single molecule,^9^

$${\text{R}}_{\text{SRS}}=\sigma_{\text{SRS}}\cdot \phi_{\text{pump}}\cdot \phi_{\text{Stokes}},$$ (3)

where R_SRS_ is the rate of stimulated Raman gain or loss (in the unit of photon ⋅ s^−1^) and *ϕ*_pump_ and *ϕ*_Stokes_ are the photon flux of the pump and Stokes beam, respectively. R_SRS_ also describes the rate of vibrational transition of the target mode during SRS. However, this equation did not explicitly account for the frequency dependence of σ_SRS_. Herein, we start with a differential form of R_SRS_ and redefine the frequency-dependent absolute SRS cross section σ_SRS_ as

$$\frac{\text{d}{\text{R}}_{\text{SRS}}}{\text{d}\omega_{\text{p}}\text{d}\omega_{\text{S}}}=\text{n}\cdot \sigma_{\text{SRS}}\left(\omega_{\text{p}}-\omega_{\text{S}}\right)\cdot \text{F}\left(\omega_{\text{p}}\right)\cdot \text{F}\left(\omega_{\text{S}}\right),$$ (4)

where ω_p_ and ω_S_ are the angular frequency of the pump and Stokes photon, respectively. F(ω_p_) and F(ω_S_) represent the *spectral* photon flux density of the pump and Stokes beam, in the unit of (photon ⋅ s^−1^ ⋅ cm^−2^ ⋅ Hz^−1^ ⋅ rad^−1^). σ_SRS_(ω_p_ − ω_S_) is a function of ω_p_ − ω_S_, centered around Ω_0_, the central frequency of the target vibrational mode. Equation (4) can be viewed to describe the SRS process for a particular solid angle dΩ, which is the case for most SRS experiments.

Integrating Eq. (4) over both ω_p_ and ω_S_, we have the integrated form as

$${\text{R}}_{\text{SRS}}=\text{n}\cdot \iint \sigma_{\text{SRS}}\left(\omega_{\text{p}}-\omega_{\text{S}}\right)\cdot \text{F}\left(\omega_{\text{p}}\right)\cdot \text{F}\left(\omega_{\text{S}}\right)\cdot \text{d}\omega_{\text{p}}\cdot \text{d}\omega_{\text{S}}.$$ (5)

In typical narrowband SRS experiments using picosecond excitation lasers, F(ω_p_) and F(ω_S_) are narrowly distributed functions, and ω_S_ ≈ ω_p_ − Ω_0_, when tuning at vibrational resonance. Under this experimental condition, the integral in Eq. (5) can be carried out and simplified into the following form:

$${\text{R}}_{\text{SRS}}=\text{n}\cdot \sigma_{\text{SRS}}\left(\Omega_{0}\right)\cdot \phi_{\text{pump}}\cdot \phi_{\text{Stokes}},$$ (6)

where σ_SRS_(Ω_0_) is the peak value of σ_SRS_(ω_p_ − ω_S_). Obviously, Eq. (6) recovers Eq. (3) introduced recently.^9^ In that study, σ_SRS_ values determined experimentally are, hence, the peak values of the corresponding σ_SRS_ (Ω_0_).

### Connecting spontaneous Raman with stimulated Raman scattering

A key task here is how to connect σ_SRS_(ω_p_ − ω_S_) with σ_Raman_. It has been proposed that spontaneous Raman scattering can be interpreted as arising from fluctuations in the vacuum zero-point energy.^24^ A parallel thought experiment could be as follows: if we were to reduce the Stokes laser power to zero, the rate of the scattering event should gravitate toward the limit of spontaneous Raman scattering rather than vanishing completely. This line of thought suggests that the effective Stokes flux shall derive from two distinct sources: the laser field from the Stokes beam and another equivalent field originating from the zero-point energy of the vacuum. Hence, we are motivated to generalize Eq. (5) as

$$\begin{array}{ll} {\text{R}}_{\text{SRS}} & =\text{n}\cdot \iint \sigma_{\text{SRS}}\left(\omega_{\text{p}}-\omega_{\text{S}}\right)\cdot \text{F}\left(\omega_{\text{p}}\right)\cdot \left[\text{F}\left(\omega_{\text{S}}\right)+{\text{F}}_{\text{vacuum}}\left(\omega_{\text{S}}\right)\right]\\ & \;\cdot \text{d}\omega_{\text{p}}\cdot \text{d}\omega_{\text{S}}, \end{array}$$ (7)

where F_vacuum_(ω_S_) is the effective spectral Stokes photon flux density that is generated from vacuum zero-point fluctuations. This treatment is in the same spirit as the photon occupation interpretation of SRS, where n_Stokes_ + 1 was used for Stokes photon creation.^13^ Equation (7) shall be to be able to describe spontaneous Raman scattering when F(ω_S_) → 0 from the external Stokes beam. In this limit, it becomes

$${\text{R}}_{\text{SRS}}=\text{n}\cdot \iint \sigma_{\text{SRS}}\left(\omega_{\text{p}}-\omega_{\text{S}}\right)\cdot \text{F}\left(\omega_{\text{p}}\right)\cdot {\text{F}}_{\text{vacuum}}\left(\omega_{\text{S}}\right)\cdot \text{d}\omega_{\text{p}}\cdot \text{d}\omega_{\text{S}},$$ (8)

which describes spontaneous Raman scattering from the perspective of SRS induced by vacuum field. An implicit assumption here is that quantum pathways probed in SRS are equally accessible through the spontaneous Raman, which is often true for ground-state SRS far from electronic resonance.^13^ In a typical spontaneous Raman experiment, a continuous-wave pump laser [i.e., narrowband excitation profile for F(ω_p_)] is used. Equation (8) then becomes

$${\text{R}}_{\text{SRS}}=\text{n}\cdot \phi_{\text{pump}}\cdot \int \sigma_{\text{SRS}}\left(\omega_{\text{p}}-\omega_{\text{S}}\right)\cdot {\text{F}}_{\text{vacuum}}\left(\omega_{\text{S}}\right)\cdot \text{d}\omega_{\text{S}}.$$ (9)

As discussed above, Eq. (9) describes the rate of vibrational transition of the target mode from the perspective of SRS induced by vacuum field. This should recover Eq. (2) that also describes the same quantity from the perspective of σ_Raman_. Both are specifying a solid angle. Equating Eq. (2) with Eq. (9), we have

$$d\sigma_{\text{Raman}}=\frac{\omega_{\text{S}}}{\omega_{\text{p}}}\int \sigma_{\text{SRS}}\left(\omega_{\text{p}}-\omega_{\text{S}}\right)\cdot {\text{F}}_{\text{vacuum}}\left(\omega_{\text{S}}\right)\cdot \text{d}\omega_{\text{S}}.$$ (10)

Equation (10) provides a route of connecting spontaneous Raman and stimulated Raman cross sections, which are experimentally defined quantities by Eqs. (1) and (6), respectively, via the effective spectral photon flux density of vacuum, F_vacuum_(ω_S_), which will be determined in the following.

### Contribution from vacuum fluctuation

Next, we seek an expression of F_vacuum_(ω_S_) suitable for Raman scattering. According to classical electromagnetism, the number of modes of the electromagnetic field in free space in volume *V* in the frequency range (ω, ω + dω) in the polar coordinates is given by $dN=\frac{V}{8\pi^{3}}\frac{\omega^{2}}{c^{3}}d\omega d\Omega$ for each polarization.^25^ dΩ is an element of solid angle. Thus, the mode density (or photon density of states), $\rho_{\text{mode}}\left(\omega \right)$, per unit frequency in volume *V* is given by

$$\rho_{\text{mode}}\left(\omega \right)=\frac{dN}{d\omega }=\frac{V\omega^{2}}{8\pi^{3}{\text{c}}^{3}}d\Omega .$$ (11)

In quantum electrodynamics (QED), the ground state of vacuum consists of one virtual photon in each of these modes. In other words, each field mode is mathematically equivalent to a harmonic oscillator in its lowest energy (zero-point energy), and in the case of electromagnetic fields, a virtual photon.^26^ Hence, ρ_mode_(ω) is equivalent to the (virtual) photon density, ρ_photon_(ω), of the vacuum ground state: $\rho_{\text{photon}}\left(\omega \right)=\rho_{\text{mode}}\left(\omega \right)$. These are called virtual photons because they are non-radiative “photons” from zero-point vacuum fluctuation, which do not exist in the classical sense. Virtual photons are created in the vacuum out of nothing and then disappear again after an extremely short time. Although these transient virtual photons cannot be observed directly, they contribute measurably to the probabilities of observable events. For example, if these photons interact during their short existence with the electrons of an atom, the binding energies of the electrons shift ever so slightly (i.e., the Lamb shift). For another example, when two mirrors are placed facing each other in a vacuum, more virtual photons can exist around the outside of the mirrors than between them, generating a seemingly mysterious force (i.e., Casimir force) that pushes the mirrors together.

Now, let us consider that the molecules under Raman excitation are within an area *A* in the time duration of *τ*. The effective volume for the virtual photon will be *V* = *A c τ*. The effective spectral photon flux density (after area and time normalization) for the vacuum, F_vacuum_(ω_S_), can be calculated by

$${\text{F}}_{\text{vacuum}}\left(\omega_{\text{S}}\right)=\frac{\rho_{\text{photon}}\left(\omega \right)}{\text{A}\cdot \tau }=\frac{\text{A}\cdot \text{c}\cdot \tau \cdot \frac{\omega_{\text{S}}^{2}}{8\pi^{3}{\text{c}}^{3}}d\Omega }{\text{A}\cdot \tau }=\frac{\omega_{\text{S}}^{2}}{8\pi^{3}{\text{c}}^{2}}d\Omega ,$$ (12)

which represents the number of virtual photons, per unit frequency, crossing a unit area in a unit time.

### An Einstein-coefficient-like equation for Raman cross sections

Plugging the vacuum contribution of Eq. (12) into Eq. (10), we arrive at

$$d\sigma_{\text{Raman}}=\frac{\omega_{\text{S}}}{\omega_{\text{p}}}\int \sigma_{\text{SRS}}\left(\omega_{\text{p}}-\omega_{\text{S}}\right)\cdot \frac{\omega_{\text{S}}^{2}}{8\pi^{3}{\text{c}}^{2}}d\Omega \cdot \text{d}\omega_{\text{S}}.$$ (13)

Thanks to the narrow bandwidth of Raman spectral response, Eq. (13) can be approximated as follows. Recall that σ_SRS_(ω_p_ − ω_S_) is a sharp function centered around Ω_0_. For example, in the case of methanol C–O mode, Ω_0_ corresponds to the vibrational band centered at 1030 cm^−1^, with a full-width-at-half-maximum (FWHM) of ∼20 cm^−1^.^27^ For a typical spontaneous Raman scattering experiment, ω_p_ is located at 3.543 × 10^15^ rad ⋅ s^−1^ (equivalent to 532 nm wavelength) for a visible laser excitation, and ω_S_ is mostly ranging from 3.344 to 3.354 × 10^15^ rad ⋅ s^−1^ to cover the C–O mode. Within this range, ω_S_ squared only changes about 1% and can, therefore, be approximated as a constant. Hence, we can take the ω_S_ variable outside the integral, and Eq. (13) approximates to

$$\frac{d\sigma_{\text{Raman}}}{d\Omega }=\frac{1}{8\pi^{3}{\text{c}}^{2}}\frac{\omega_{\text{S}}^{3}}{\omega_{\text{p}}}\cdot \int \sigma_{\text{SRS}}\left(\omega_{\text{p}}-\omega_{\text{S}}\right)\cdot \text{d}\omega_{\text{S}},$$ (14)

where we have used ω_S_ = ω_p_ − Ω_0_ and the definition of differential Raman cross section dσ_Raman_/dΩ.

We next introduce the lineshape function G(ω) for the frequency dependence of σ_SRS_ around one vibrational mode. Since the absolute position of the peak is not important in the integral, the normalization requires $\int \text{G}\left(\omega_{\text{p}}-\omega_{\text{S}}\right)\cdot \text{d}\omega_{\text{S}}=1$. Then, we can rewrite Eq. (14) as

$$\frac{d\sigma_{\text{Raman}}}{d\Omega }=\frac{1}{8\pi^{3}{\text{c}}^{2}}\frac{\omega_{\text{S}}^{3}}{\omega_{\text{p}}}\cdot \int \frac{\sigma_{\text{SRS}}\left(\omega_{\text{p}}-\omega_{\text{S}}\right)}{\text{G}\left(\omega_{\text{p}}-\omega_{\text{S}}\right)}\cdot \text{G}\left(\omega_{\text{p}}-\omega_{\text{S}}\right)\cdot \text{d}\omega_{\text{S}}.$$ (15)

Note that G(ω) has the same frequency dependence as σ_SRS_(ω). Hence, $\frac{\sigma_{\text{SRS}}\left(\omega_{\text{p}}-\omega_{\text{S}}\right)}{\text{G}\left(\omega_{\text{p}}-\omega_{\text{S}}\right)}$ is just a constant equal to $\frac{\sigma_{\text{SRS}}\left(\Omega_{0}\right)}{\text{G}\left(\Omega_{0}\right)}$. Equation (15) then becomes

$$\begin{array}{ll} \frac{d\sigma_{\text{Raman}}}{d\Omega } & =\frac{1}{8\pi^{3}{\text{c}}^{2}}\frac{\omega_{\text{S}}^{3}}{\omega_{\text{p}}}\cdot \frac{\sigma_{\text{SRS}}\left(\Omega_{0}\right)}{\text{G}\left(\Omega_{0}\right)}\cdot \int \text{G}\left(\omega_{\text{p}}-\omega_{\text{S}}\right)\cdot \text{d}\omega_{\text{S}}\\ & =\frac{\omega_{\text{S}}^{3}}{8\pi^{3}{\text{c}}^{2}\omega_{\text{p}}\text{G}\left(\Omega_{0}\right)}\cdot \sigma_{\text{SRS}}\left(\Omega_{0}\right), \end{array}$$ (16a)

where we have used the normalization condition of G(ω). For simplicity, here, we assume the scattering is isotropic, and integrating dσ_Raman_/dΩ over all spatial angles and considering the two polarizations of light gives the total cross section,

$$\sigma_{\text{Raman}}=\frac{\omega_{\text{S}}^{3}}{\pi^{2}{\text{c}}^{2}\omega_{\text{p}}\text{G}\left(\Omega_{0}\right)}\cdot \sigma_{\text{SRS}}\left(\Omega_{0}\right).$$ (16b)

If we model the Raman lineshape function G(ω) by a normalized Lorentzian profile $L\left(\tilde{\nu }\right)=\frac{1}{\pi }\frac{\frac{1}{2}\Gamma }{{\left(\tilde{\nu }-{\tilde{\nu }}_{0}\right)}^{2}+{\left(\frac{1}{2}\Gamma \right)}^{2}}$, where Γ is the FWHM of the peak, the peak value $\text{G}\left(\Omega_{0}\right)={\left.L\left(\tilde{\nu }\right)\right|}_{\tilde{\nu }={\tilde{\nu }}_{0}}=\frac{2}{\pi \Gamma }$, then Eq. (16b) becomes

$$\sigma_{\text{Raman}}=\frac{\omega_{\text{S}}^{3}\Gamma }{2\pi {\text{c}}^{2}\omega_{\text{p}}}\cdot \sigma_{\text{SRS}}\left(\Omega_{0}\right),$$ (16c)

which is a simple practical form of Eq. (16b). It is interesting to see how the peak width Γ plays a role here. Intuitively, Γ controls the spectral window of the vacuum photon modes that can contribute.

The factors in front of σ_SRS_(Ω_0_) together display a unit of photon/(s ⋅ cm^2^), suggesting a physical quantity of photon flux. Hence, we are prompted to group them into an effective photon flux arising from the vacuum field fluctuation,

$$\sigma_{\text{Raman}}=\sigma_{\text{SRS}}\left(\Omega_{0}\right)\cdot \phi_{\text{vacuum}}\text{where }\phi_{\text{vacuum}}\equiv \frac{\omega_{\text{S}}^{3}\Gamma }{2\pi {\text{c}}^{2}\omega_{\text{p}}}.$$ (16d)

Equation (16) is a key result that relates normal Raman cross section with absolute SRS cross section, which are experimentally defined by Eqs. (1) and (6), respectively. It carries the same spirit of Einstein’s A and B coefficients relating spontaneous emission rate with stimulated emission.

In summary, we have established a connection between spontaneous Raman and stimulated Raman cross sections without explicitly referring to full quantum mechanics. The general methodology adopted here is similar to Einstein’s derivation of the A and B coefficients: we started from the experimental definition of two sets of cross sections in the form of transition rate, and we identified a logical link between the two formulas to determine their relationship. In Einstein’s derivation, the connection is established by comparing to Planck’s blackbody radiation law and microscopic reversibility, and the linkage we used here is how the vibrational transition rate of SRS should recover that of regular Raman when the external laser field vanishes.

It is constructive to compare our result to the Füchtbauer–Ladenburg equation, which was introduced long time ago for treating fluorescence in the literature of atomic physics,^28,29^

$$\frac{1}{\tau_{\text{rad}}}=\frac{8\pi }{{\text{c}}^{2}}\int \nu^{2}\sigma_{\text{em}}\left(\nu \right)d\nu ,$$ (17)

where σ_em_(ω) is the stimulated emission cross section and τ_rad_ is the lifetime of the upper level. The Füchtbauer–Ladenburg equation, which is often regarded as a generalized form of Einstein’s coefficients, is usually not evaluated in close forms. This is due to the relatively large bandwidth of electronic transition and the term *ν*^2^ is no longer a constant in the integration.^28^ In contrast, Eq. (16b) gives a simple close form, thanks to the narrow bandwidth of Raman spectral response. In fact, if we replace the angular frequency ω with linear frequency *ν*, we shall transform Eq. (13) into the following form (Note S1):

$$\sigma_{\text{Raman}}=\frac{8\pi }{{\text{c}}^{2}}\frac{\nu_{S}}{\nu_{\text{p}}}\cdot \int {\nu_{\text{S}}}^{2}\cdot \sigma_{\text{SRS}}\left(\nu_{\text{p}}-\nu_{\text{S}}\right)\cdot \text{d}\nu_{\text{S}}.$$ (18)

This form of Eq. (18) is almost identical to the Füchtbauer–Ladenburg equation, supporting the validity of our equation. It is interesting to note that a coherent process (such as stimulated emission or stimulated Raman) is connected to an incoherent process (such as fluorescence or spontaneous Raman), which can be viewed from the perspective of a single molecule.

### Comparing theoretical prediction with experimental measurements

We now compare the prediction of Eq. (16c) with experimentally measured Raman cross sections. First, we modify Eq. (16c) into a more ready-to-use format for experimentalists. For example, ω_p_ is replaced by wavelength *λ*_*p*_, and Ω_0_ and Γ are replaced by wavenumbers *ṽ*_0_ and *ṽ*_Γ_. Then, we obtain (Note S2)

$$\sigma_{\mathrm{Raman}}=4\pi^{2}c\cdot \frac{\lambda_{p}{\tilde{\nu }}_{\Gamma }}{\lambda_{S}^{3}}\cdot \sigma_{\mathrm{SRS}}.$$ (19)

Assuming *λ*_*p*_ and *λ*_*S*_ are in nm, *ṽ*_Γ_ is with a unit of cm^−1^, and σ_SRS_ is in GM, we then have numerically (Note S2)

$$\sigma_{\text{Raman}}=1.18\times 10^{-24}\cdot \frac{\lambda_{p}{\tilde{\nu }}_{\Gamma }}{\lambda_{S}^{3}}\cdot \sigma_{\mathrm{SRS}}\;\text{c}{\text{m}}^{2}.$$ (20)

The methanol C–O *ṽ*_0_ = 1030 cm^−1^ mode is a commonly used standard for Raman scattering, and its cross section has been carefully measured and documented in the literature. In particular, σ_SRS,C_–_O_ = 0.04 GM = 4 × 10^−52^ cm^4^ ⋅ s^−1^ photon^−1^ has been recently measured as the peak value of σ_SRS_ (Ω_0_) in a narrowband SRS experiment.^9^ The SRS pump excitation and Stokes scattering wavelengths are at *λ*_*p*_ = 960 nm and *λ*_*S*_ = 1064 nm, respectively. Γ is experimentally estimated to be corresponding to ∼20 cm^−1^.^27^ Plugging these parameters, we obtain

$$\sigma_{\text{Raman},\text{predicted}}=7.5\times 10^{-31}\;{\text{cm}}^{2}.$$ (21)

**FIG. 1.**
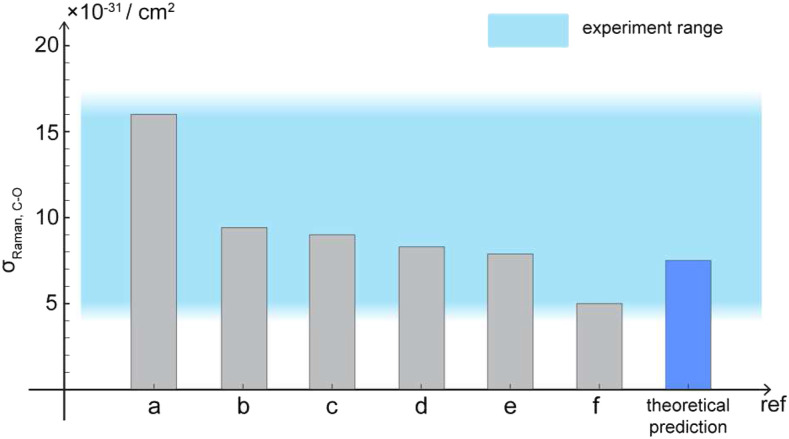
Comparison between theory and experiments. The methanol C–O Raman stretching mode cross section has reported values of (a)^30^ 1.6 × 10^−30^ cm^2^, (b)^31^ 9.7 × 10^−31^ cm^2^, (c)^18^ 9.1 × 10^−31^ cm^2^, (d)^32^ 8.4 × 10^−31^ cm^2^, (e)^33^ 7.9 × 10^−31^ cm^2^, and (f)^34^ 5.1 × 10^−31^ cm^2^ after conversion for pump laser frequency. Raman cross section predicted from Eq. (21) is about 7.5 × 10^−31^ cm^2^, which shows good agreement with measured results.

Meanwhile, the spontaneous Raman cross section has been experimentally determined by several reports to range between 5.1 × 10^−31^ and 1.6 × 10^−30^ cm^2^. We have summarized the results in Fig. 1. Results are converted into an excitation profile at 960 nm using the $\omega_{p}\omega_{S}^{3}$ dependency, and differential cross sections are integrated to total cross sections using the following equation described in the literature:^18^

$$\sigma_{R}=\frac{8\pi }{3}\frac{1+2\rho }{1+\rho }\frac{\partial \sigma }{\partial \Omega_{\|⊥}},$$ (22)

where *ρ* = 0.21 is the depolarization ratio measured for methanol C–O stretching and $\frac{\partial \sigma }{\partial \Omega_{\|⊥}}$ is the differential cross-section measured in both the parallel and perpendicular directions. Remarkably, the Raman cross section predicted from Eq. (21) closely aligns with the experimental results (Fig. 1). Taking the inhomogeneous broadening effect into consideration, we have applied the same method to the Gaussian profile, and the result is still within the range of the reported values (Note S3). Note that these two sets of experiments were performed in rather different instruments and the cross sections were calculated using other parameters (such as concentration, focal volume, laser power, collection efficiency, and slit width) with varying degrees of uncertainty. Hence, this agreement is considered to be satisfactory under current measurement efforts. This result could be further improved by rigorous calibration of SRS instrument errors coming from detector response, optical alignment, and electronic noise. Therefore, the experimental measurement results support the validity of Eq. (16).

## DISCUSSIONS AND CONCLUSIONS

### Raman scattering as a vacuum fluctuation-induced effect

Contemporary physicists, when asked to give a physical explanation for the occurrence of spontaneous emission, generally invoke the vacuum electromagnetic field.^26^ This view was first introduced by Weisskopf in 1935^35^ and was mentioned later on multiple occasions.^36,37^ It was subsequently adopted in Raman spectroscopy by interpreting spontaneous Raman scattering as arising from fluctuations in the vacuum zero-point energy.^24^ However, a quantitative relation between σ_Raman_ and σ_SRS_ did not exist in the literature, to the best of our knowledge. This is likely because σ_SRS_ has not been explicitly defined and measured for molecule systems until very recently. Now, one can have a more comprehensive list of effects that are the physical consequences of the fluctuating vacuum field. These effects include phenomena such as spontaneous emission, spontaneous Raman, Casimir force, van der Waals forces, as well as the Lamb shift. To some extent, this further proves the significance of vacuum in the understanding of nature and possibly opens the door for connecting Raman and quantum-vacuum-related studies, such as quantum-entangled Raman^38^ and relativistic Raman scattering.

### Why do Raman cross sections appear so small?

Raman scattering has been acknowledged in textbooks and reviews for decades as an extremely weak process.^39^ The values of σ_Raman_ (10^−30^ to 10^−28^ cm^2^ for small chemical bonds) turn out to be many orders of magnitude smaller in comparison to other molecular spectroscopies, such as UV–vis absorption cross sections (10^−16^ to 10^−15^ cm^2^) or infrared cross sections (10^−19^ to 10^−17^ cm^2^) of similar bonds. Even with the boost of the electronic resonance effect in resonance Raman spectroscopy (σ_Raman_ can increase up to 10^−24^ to 10^−23^ cm^2^), the gap is still as wide as many orders of magnitude.^12,40,41^

Despite the shared unit of cm^2^ for cross section, linear absorption and Raman scattering are two very different processes. Both UV–vis and infrared cross sections describe absorption processes, and all absorptions are stimulated transitions by nature. However, Raman scattering naturally includes the spontaneous emission process. The participation of vacuum in the spontaneous emission process underlies the differences. Fortunately, Eq. (16d) suggests that σ_Raman_ is not an intrinsic response of the molecule but rather a special case of SRS where the vacuum field instead of an external laser beam is providing the Stokes photon flux. The vacuum-induced contribution is being absorbed into σ_Raman_. Quantitatively, this contribution can be treated as an effective vacuum photon flux, as explicitly shown in Eq. (16d). We can easily estimate *ϕ*_vacuum_ from Eq. (16d) using the above-calculated values for methanol C–O bond,

$$\begin{array}{ll} \phi_{\text{Stokes},\text{vacuum}} & =\frac{{\left(1.772\times 10^{15}\;\text{rad}\cdot {\text{s}}^{-1}\right)}^{3}\cdot 3.8\times 10^{12}\;\text{rad}\cdot {\text{s}}^{-1}}{2\pi \;{\text{rad}}^{3}\cdot {\left(3\times 10^{10}\;\text{cm}\cdot {\text{s}}^{-1}\right)}^{2}\times 1.963\times 10^{15}\;\text{rad}\cdot {\text{s}}^{-1}}\\ & =2\times 10^{21}\text{photon}\;\text{c}{\text{m}}^{-2}\cdot {\text{s}}^{-1}. \end{array}$$ (23)

We can further gain insight by converting this value to an equivalent free-space laser that can produce such a level of photon flux. We imagine that this level of photon flux was to be produced by focusing a propagating real-space laser down to a diffraction-limited focal spot under a microscope. Assuming a *λ* = 532 nm CW laser beam with a diameter *d* = 600 nm, we would get an effective laser power of

$$\begin{array}{ll} {\text{P}}_{\text{vacuum}} & =\frac{1}{4}\pi d^{2}\cdot \phi_{\text{Stokes},\text{vacuum}}\cdot \text{h}\frac{\text{c}}{\lambda }\\ & =\left[\frac{\pi }{4}\times {\left(6\times 10^{-7}m\right)}^{2}\right]\cdot 2\times 10^{25}\text{photon}\cdot {\text{m}}^{-2}\cdot {\text{s}}^{-1}\\ & \;\times \left[6.626\times 10^{-34}\text{J}\cdot \text{s}\cdot \frac{3\times 10^{8}m\cdot s^{-1}}{5.32\times 10^{-7}m}\mathrm{photo}n^{-1}\right]\\ & =2\times 10^{-6}\text{W}=2\;\mu W. \end{array}$$ (24)

In a sense, this value can be regarded as the total zero-point energy (off by a factor of 2) of the virtual vacuum photon that drives the Raman scattering of methanol C–O bond in a microscope experimental setting. In other words, one can think of Raman scattering as being jointly excited by an external pump CW laser and a CW Stokes laser of a few microwatts (provided by the vacuum fluctuation) in a two-photon third-order nonlinear manner. In comparison, modern microscopy experiments (such as two-photon excited absorption) typically employ tens of milliwatts of laser power and 100 fs short pulses to induce stimulated absorption. One can estimate the corresponding photon flux during the pulse to be orders of magnitude (∼10^7^) higher than the value in Eq. (23). Thus, the feeble vacuum-field-induced photon flux is the underlying mechanism of the apparently weak Raman response of σ_Raman_.

### Intrinsically strong molecular response of stimulated Raman cross sections

Contrary to common belief, the effect of vacuum might not be constant in all circumstances. Indeed, in cavity QED,^42^ the rate of spontaneous emission could be controlled depending on the boundary conditions of the surrounding vacuum field. The possible enhancement or inhibition of the spontaneous emission rate is known as the Purcell effect.^43^ Similar effects have been observed in Raman scattering, although several decades later. In 1993, Cairo *et al.* put a Raman medium (C_6_H_6_) in a cavity and observed that, as spontaneous emission, it is possible to enhance or inhibit spontaneous Raman scattering for a specific Raman line, just by spectral tuning of the cavity.^20^ Since then, more similar observations have been made.^21–23^

Having decoupled the contribution from the vacuum field that depends on the boundary conditions of the surrounding environment, Eq. (16) suggests that σ_SRS_ should be a vacuum-field-independent, truly molecular intrinsic quantity for describing Raman response when compared to σ_Raman_. This view shall hold for both stimulated Raman processes and spontaneous Raman processes. σ_SRS_ can be either converted from regular Raman cross sections theoretically or directly determined by SRS spectroscopy experiments (as recently done for many Raman-active probes). An important consequence is that σ_SRS_ carries a unit of Göppert–Mayer (1 GM = 10^−50^ cm^4^ ⋅ s photon^−1^), the same unit used in the two-photon absorption. This allows meaningful comparison in this new axis.

**FIG. 2.**
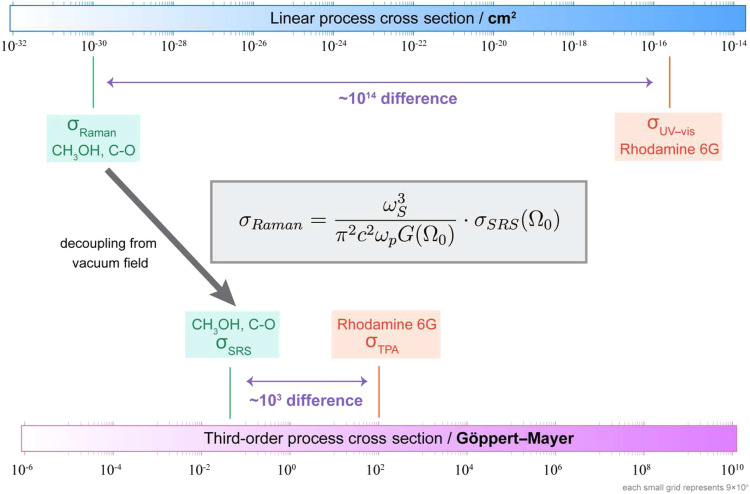
Intrinsically strong stimulated Raman cross section: a case study. Normal Raman scattering has always been considered an extremely weak process due to the 13–15 orders of magnitude of difference in cross sections when compared to other processes such as linear absorption. However, after decoupling the effect of vacuum field, the molecular-intrinsic SRS cross section is much closer in value to the counterparts of two-photon absorption (TPA), with only three orders of magnitude difference. Shown in the figure are σ_Raman_ and σ_SRS_ for methanol C–O stretching mode at 1030 cm^−1^, and the peak UV–vis absorption cross section σ_UV–vis_ and two-photon absorption cross section σ_TPA_ for Rhodamine 6G (Table S1). In essence, this tells us (σ_UV–vis_/σ_Raman_)/(σ_TPA_/σ_SRS_) = 10^11^ for this case.

Figure 2 shows the comparison between the methanol C–O bond and Rhodamine 6G as a case study. Rhodamine dye is chosen here as it is a well-documented model compound. σ_Raman_ of methanol C–O bond is 10^14^ times smaller than the UV–vis absorption cross section of Rhodamine 6G, which is essentially the reason for criticizing Raman being an extremely weak process in many textbooks. However, the vacuum-decoupled σ_SRS_ is only 10^3^ times away from the two-photon absorption cross section σ_TPA_ for Rhodamine 6G. Hence, this example highlights that molecule-intrinsic Raman response is not weak after all—the new perspective has narrowed the gap of conventional comparison of cross sections by over 10^10^ folds. In fact, previous simultaneous measurements on SRS, TPA, and other pump–probe processes on the same molecules (such as melanin) have hinted that the SRS response has an appreciable magnitude.^44^ Recent invention of stimulated Raman photothermal microscopy also implies strong intrinsic response.^45^ Only after being driven by the weak vacuum field [Eq. (23)], the resulting σ_Raman_ becomes a small quantity.

### A phenomenological framework for connecting different photonic processes

**FIG. 3.**
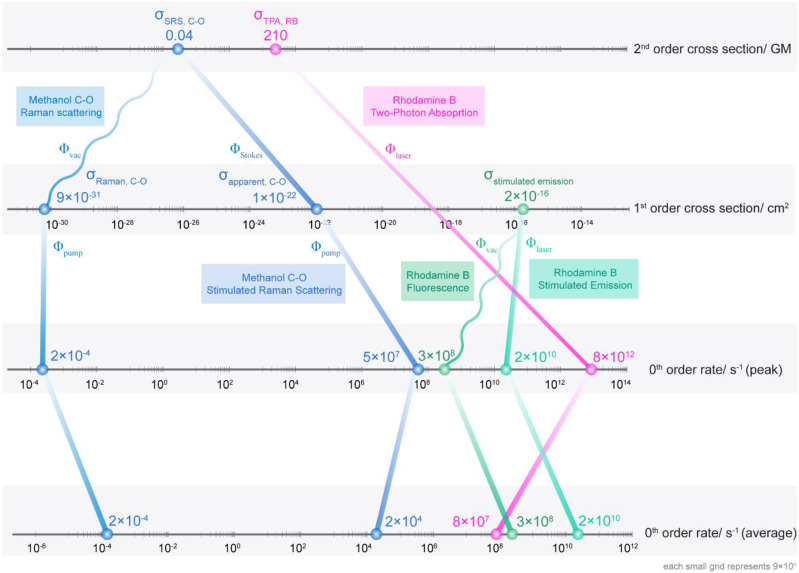
Connecting different photonic processes via cross sections of varying orders. Data points for two representative bonds/molecules (methanol C–O and Rhodamine B) and five photonic processes [including spontaneous Raman scattering, stimulated Raman scattering, two-photon absorption, fluorescence (i.e., spontaneous emission) and stimulated emission] are illustrated. These processes shall be connected with photon flux, either from vacuum (wavy lines connected to Raman scattering and fluorescence) or from external laser beams (straight lines). Only data points without a connection to a higher order quantity represent the intrinsic property of the molecule/bond. The peak zeroth rate and average zeroth rate can be connected via the duty cycle of the laser. Detailed calculations are shown in Note S4.

We shall borrow the concept of reaction order from chemical kinetics to organize common optical processes into three categories (Fig. 3), each with a different unit. Zeroth-order processes have a unit of s^−1^, representing the rate that we can directly measure in experiments. First-order photonics have a unit of cm^2^ as is used in linear absorption, stimulated emission, and spontaneous Raman scattering. Second-order processes with a unit of GM are less common, the most popular of which are SRS and two-photon absorption.

These different orders of reactions can be related through the multiplication of proper molecular cross sections by a photon flux or several photon fluxes, which can derive either from vacuum (wavy lines) or from external laser beams (straight lines). Take Raman scattering for example, if we multiply the second-order σ_SRS_ by the effective vacuum photon flux *ϕ*_vacuum_, we shall get σ_Raman_, as shown in Eq. (16b). On the other hand, if we multiply σ_SRS_ by the photon flux from an external Stokes laser beam *ϕ*_Stokes_, we shall get the apparent stimulated Raman cross section σ_apparent_, defined as σ_apparent_ = σ_SRS_ ⋅ *ϕ*_Stokes_ previously.^9^ Further multiplication of the first-order σ_Raman_ or σ_apparent_ by the pump photon flux *ϕ*_pump_ will lead to the zeroth-order rate in the unit of s^−1^, which can be experimentally measured. Similarly, if we multiply the stimulated emission cross section σ_se_ of Rhodamine B by *ϕ*_vacuum_, we would obtain the rate of transition in fluorescent emissions, usually expressed in the inverse form of lifetime (∼3 ns). A product of σ_se_ and *ϕ*_laser_ would result in stimulated emission rate, a parameter commonly used in predicting stimulated emission depletion (STED) efficiency. Two-photon absorption (TPA) transitions are different in the sense that a direct transition from the second order to the zeroth order was achieved at once by multiplication σ_TPA_ with the squared value of *ϕ*_laser_. Note that direct multiplication of cross section and photon flux would result in peak transition rate (i.e., rate within the laser pulse), and the average rate can be calculated by multiplying the peak rate by the duty cycle of the laser (Note S4). These results are organized in Fig. 3.

Based on this figure, it can be concluded that only those data points that are not connected to a higher order quantity represent the molecule or bond’s true intrinsic property. Thus, they should only be compared to quantities located on the same axis. In this sense, we should only compare SRS with TPA and compare absorption with stimulated emission. Despite the shared unit of cm^2^, σ_Raman_ and σ_abs_ lie on very different grounds in terms of photonic nature because σ_Raman_ contains the contribution from the vacuum field, whereas σ_abs_ does not. This framework can also be expanded to even higher order processes such as three-photon absorption cross sections, which should be a third-order process.

## SUPPLEMENTARY MATERIAL

See the supplementary material for the details of cross sections of Rhodamine 6G, calculation in linear frequency, calculation of predicted experimental Raman cross sections, calculation of cross sections under Gaussian profile, and calculation of cross sections and rates.

## Data Availability

The data that support the findings of this study are available from the corresponding author upon reasonable request.
